# Aberrant activation of the human sex-determining gene in early embryonic development results in postnatal growth retardation and lethality in mice

**DOI:** 10.1038/s41598-017-04117-6

**Published:** 2017-06-23

**Authors:** Tatsuo Kido, Zhaoyu Sun, Yun-Fai Chris Lau

**Affiliations:** Department of Medicine, VA Medical Center, and Institute for Human Genetics, University of California, San Francisco, San Francisco, California USA

## Abstract

Sexual dimorphisms are prevalent in development, physiology and diseases in humans. Currently, the contributions of the genes on the male-specific region of the Y chromosome (MSY) in these processes are uncertain. Using a transgene activation system, the human sex-determining gene *hSRY* is activated in the single-cell embryos of the mouse. Pups with *hSRY* activated (hSRY^ON^) are born of similar sizes as those of non-activated controls. However, they retard significantly in postnatal growth and development and all die of multi-organ failure before two weeks of age. Pathological and molecular analyses indicate that hSRY^ON^ pups lack innate suckling activities, and develop fatty liver disease, arrested alveologenesis in the lung, impaired neurogenesis in the brain and occasional myocardial fibrosis and minimized thymus development. Transcriptome analysis shows that, in addition to those unique to the respective organs, various cell growth and survival pathways and functions are differentially affected in the transgenic mice. These observations suggest that ectopic activation of a Y-located *SRY* gene could exert male-specific effects in development and physiology of multiple organs, thereby contributing to sexual dimorphisms in normal biological functions and disease processes in affected individuals.

## Introduction

Sexual dimorphisms are prevalent in normal development and physiology, such as brain structures, cognition, and blood pressure phenotypes^1–6^; and pathogeneses of diseases, including neurodevelopmental diseases, such as autism and Hirschsprung disease; cognitive disorders, such as schizophrenia; neurodegenerative diseases, such as Alzheimer and Parkinson diseases; pulmonary disorders, such as bronchopulmonary dysplasia; and metabolic and hepatic diseases, such as non-alcoholic fatty liver disease; and cardiovascular diseases, such as cardiomyopathies and hypertension^7–20^. Currently, the mechanisms associated with such sex differences have not been fully investigated. Sex hormones and their receptors could have significant differential effects in these developmental, physiological and pathogenic processes^3, 7, 12, 21–23^. At present the contributions of genes on the male-specific region of the Y chromosome (MSY) to sex differences in development, physiology and diseases are uncertain. Recent sequencing studies on the mammalian Y chromosomes showed that most MSY genes have homologues on the X chromosome, and potentially share similar functions in transcription, translation, chromatin modification, RNA processing and protein stability^24^. Among the 17 human MSY genes, four, i.e. *SRY*, *TSPY*, *RBMY*, and *HSFY*, had diverged significantly from their corresponding X homologues, i.e. *SOX3*, *TSPX*, *RBMX and HSFX* respectively, and evolved to serve male-specific functions in sex determination and sperm production. Although they are mostly expressed in the testis, their expressions in non-gonadal tissues have been well-documented^25–33^. The sex-determining *SRY* gene, in particular, serves critical function in determining the male fate of the sex organ during embryogenesis, and is the most critical gene in normal male development. Hence, an aberrant activation of a MSY-located *SRY* in non-gonadal cells could disrupt/modify the normal gene regulatory programs, thereby exerting male-specific effects on the developmental, physiological and/or pathological processes of the affected cells/tissues^24–29, 31^.

SRY is the founder of a family of 20 transcription factors, harboring an SRY-related HMG box (SOX)^34^. In particular, the *SOXE* genes, i.e. *SOX9*, *SOX10* and *SOX8*, play key roles beyond the initial *SRY* actions in male sex differentiation as well as development and differentiation of numerous non-gonadal organs, including the central and peripheral nervous systems, liver, pancreas and bile duct, chondrocytes and cartilages, prostate gland, inner ear, and aorta^35–44^. They share homology with SRY only at their DNA-binding SOX domain, but diverge in their flanking sequences. Previously, we showed that SRY and SOX9 share close to half of their respective targets in the Sertoli cells during sex determination, and can differentially regulate each other’s target genes^45^. Based on these observations, we hypothesize that an ectopically expressed *SRY* in non-gonadal tissues could compete with *SOX9*/*SOXs*, and possibly other transcription factors, in their gene regulatory programs, thereby exerting male-specific effects and sexual dimorphisms in tissues/diseases in spatiotemporal manners.

In order to determine the global effects of *SRY* ectopic expression in development and physiology, we have established a Cre-LoxP transgene activation system, and evaluated the consequences of ectopic activation of the human *SRY* gene in transgenic mice. Our results show that pups with ectopic *SRY* activation (hSRY^ON^) at single-cell embryonic stage are born alive in similar size as those of non-transgenic or *Ddx4-Cre* transgenic littermates, but they retard significantly in growth and all die postnatally before two-weeks of age. Pathohistotology analyses show severe impairments in development of the heart, lung, liver and brain in ﻿h﻿SRY^ON^ animals, resulting in heart fibrosis, retarded alveologenesis in the lung, impaired neurogenesis in the brain, and hepatic steatosis. These defects apparently lead to multi-organ failures and postnatal death. Transcriptome analysis shows that unique sets of genes are differentially affected by ectopic *SRY* expression, which disturbs various canonical pathways, biological functions and signaling processes in the respective organs. Our results support the hypothesis that when ectopically expressed, *SRY* could differentially affect the normal development and physiology of somatic tissues/organs, and contribute to the pathogeneses of numerous diseases with significant male preferences.

## Results

### Establishment of a Cre-LoxP transgene activation system in the mouse

**Figure 1:**
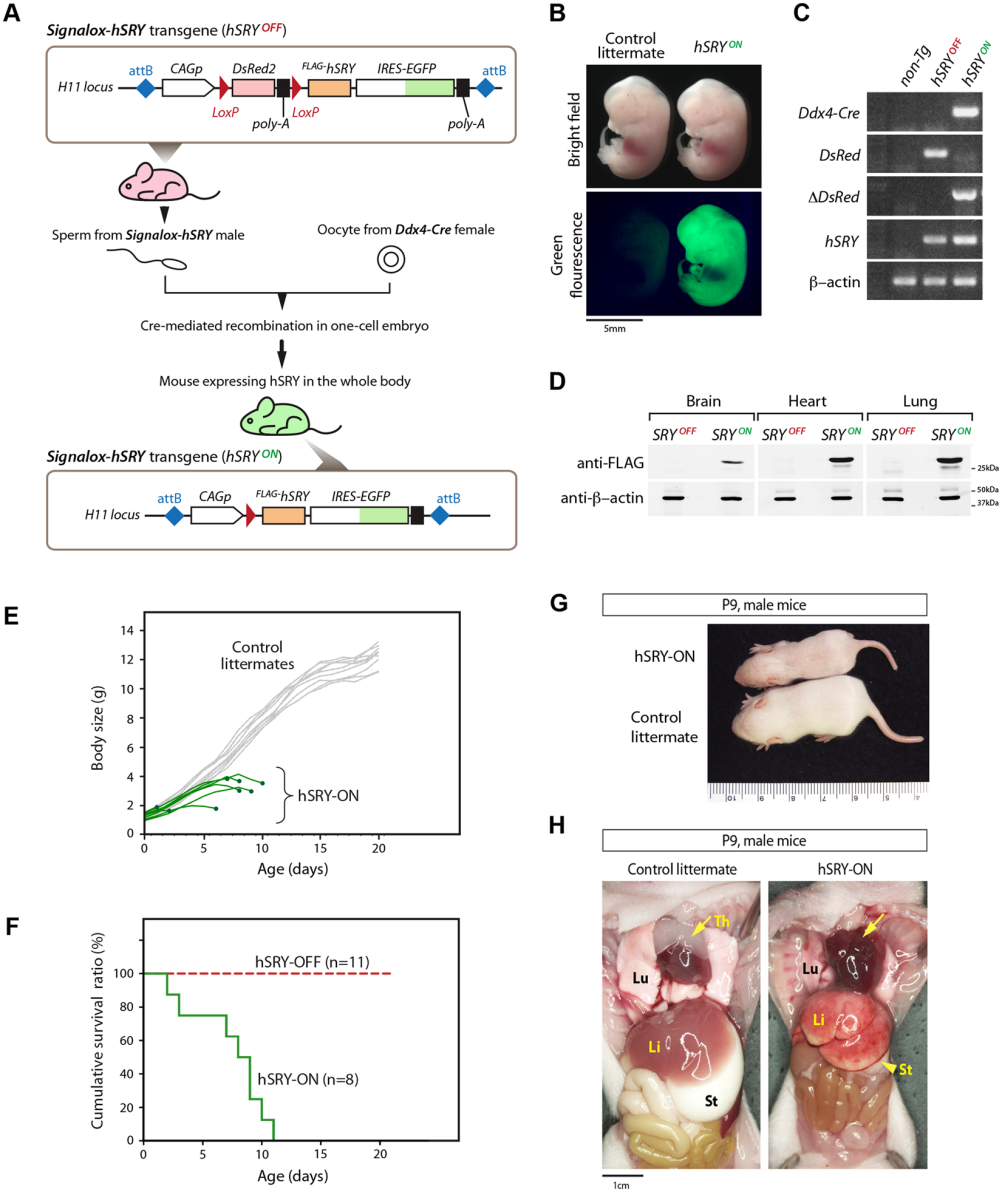
The Cre-LoxP transgene activation strategy for aberrant expression of the human *SRY* and growth retardation in transgenic mice. (**A**) Responder gene harboring human *SRY-IRES-EGFP* expression cassette (top box), capable of being activated with a Cre recombinase, i.e. from oocyte of female *Ddx4-Cre* transgenic mice (middle), resulting in activation of *SRY-IRES-EGFP* cassette (bottom box). (**B**) Co-expression of the *EGFP* gene in a E12.5 hSRY-ON embryo. (**C**) PCR analysis of non-transgenic, non-recombined and recombined *Signalox-hSRY*, showing reposition of *SRY-IRES-EGFP* under the *CAG* promoter. (**D**) Western blot analysis of protein lysates of brain, heart and lung from newborn, showing the absence and presence of FLAF-tagged human SRY protein in SRY^OFF^ and SRY^ON^ pups respectively. (**E**) Changes in body size of hSRY-ON and control littermates with age (in days). (**F**) Survival of hSRY-OFF and hSRY-ON pups with age (in days). (**G**) Example of the size of hSRY-ON and hSRY-OFF pups at P9 age. (**H**) Necropsy at P9 stage, showing lack of milk in the stomach (St) and digestive tract, absence of the thymus (Th, yellow arrows), and discolored lung (Lu) and liver (Li) in hSRY-ON animal (right), as compared to the control littermate (left).

To investigate the effects of an ectopically expressed human *SRY* transgene in mouse development and physiology, we have developed a Cre-LoxP transgene activation system^46^, which consists of a responder and an activator transgenic mouse lines. The responder line harbors a bicistronic human *SRY-IRES-EGFP* transgene, which is normally silent but can be activated with a *Cre* recombinase activator (Fig. 1A, top box). Under normal conditions, a red fluorescent protein gene, *DsRed2*, is expressed under the direction of the strong actin *CAG* promoter, while the FLAG-tagged human *SRY-IRES-EGFP* gene is silent. However, in the presence of an activator, i.e. a *Cre* recombinase, the *DsRed2* gene is deleted while the *SRY-IRES-EGFP* coding sequence is repositioned immediately after the *CAG* promoter, thereby activating the ^*FLAG*^ *hSRY* and *EGFP* simultaneously (hSRY^ON^, Fig. 1, bottom box).

To establish this transgene activation system, we had generated a transgenic mouse responder line harboring a single-copy integration of the *Signalox-hSRY* cassette at the *H11* locus on chromosome 11 of the mouse using the TARGATT homologous recombination technique^47^. The *Ddx4*-*Cre* transgenic mouse line^48^ was used as the activator. *Ddx4* (*Vasa*) is a RNA binding helicase essential for germ cell development. *Ddx4-Cre* transgene is expressed specifically in germ cells as early as E15 day of gestation, and throughout postnatal germ cell lineage of both sexes. *Ddx4-*directed Cre recombinase is capable of mediating a recombination of sequences flanked by LoxP sites with greater than 95% efficiency^48^. However, due to differences in cytoplasmic contents between sperms and oocytes, Cre recombinase protein is efficiently transferred to the fertilized single-cell embryos from the oocyte, but not the sperm^48^. Hence, when a female *Ddx4-Cre* mouse is crossed with a male *Signalox-hSRY* mouse, highly efficient recombination takes place in the early embryo, thereby activating the *hSRY* in the single-cell embryonic stage, irrespective the presence of the *Ddx4-Cre* transgene. The animals resulting from such crosses are hereto designated as hSRY^ON^ (or hSRY-ON) mice. Littermates harboring either the *Ddx4*-*Cre* transgene only or none of the parental transgenes (non-transgenic) are used as controls. Since under the natural conditions, only males have *SRY* and any ectopic *SRY* activation in the disease conditions will occur in males, we have focused on male hSRY^ON^ offspring in this experimental model.

All hSRY^ON^ mouse embryos and pups showed co-expression of EGFP, detectable by direct visualization of the green fluorescence, but not in their littermate controls (Fig. 1B). PCR analysis of tail DNAs with specific primer sets corresponding to recombined and non-recombined *Signalox-hSRY* transgene showed that successful Cre-mediated recombination did occur in the genomes of hSRY^ON^ embryos (Fig. 1C). The expression of the activated ^*FLAG*^ *hSRY* could be detected readily with western blotting in total protein lysates of the brain, heart, and lung of newborn (P0) hSRY^ON^ pups, but not those from age-matched non-recombined controls (SRY^OFF^, Fig. 1D). These initial results showed that *Signalox-hSRY* was successfully recombined in the single-cell embryos by the Cre recombinase transferred from the oocytes of *Ddx4-Cre* transgenic mother and the hSRY and the EGFP tracer were co-activated and co-expressed in the hSRY^ON^ animals under the spatiotemporal regulation of the actin *CAG* promoter.

### Ectopic activation of human SRY in single-cell embryos results in abnormalities in multiple organs and postnatal lethality

The hSRY^ON^ pups and control littermates were born of similar body size in the same litters (Fig. 1E). However, selected hSRY^ON^ pups began to die as early postnatal day 2, and none lived beyond two-weeks of age (Fig. 1E,F). The hSRY^ON^ pups grew notably slower than their littermate controls (Fig. 1E and G). Necropsy analysis showed that hSRY^ON^ pups had significant abnormalities, including lack of milk in their stomachs and digestive tract, discolored and bloody-looking liver and lung, and occasional absence of the thymus (Fig. 1H). Time-lapse video recording showed that hSRY^ON^ pups had minimal innate suckling activities, as compared to littermate controls, and was likely responsible for milk deficiency in their stomachs and digestive tracts. The absence or minimized thymus suggests that the development of this organ was impaired, potentially affecting the T-cell differentiation and T-cell repertoire development important for the immune system^49–51^. Bi-transgenic offspring from the reverse crosses between male *Ddx4-Cre* and female *Signalox-SRY* mice showed *hSRY* recombination and expression in the germ cell lineage, and grew normally to adulthood as their littermates (Supplemental Fig. 1). These observations suggest that early activation of the human SRY transgene in non-gonadal cells could be important in the growth retardation and postnatal lethality phenotypes observed in the hSRY^ON^ animals.

**Figure 2:**
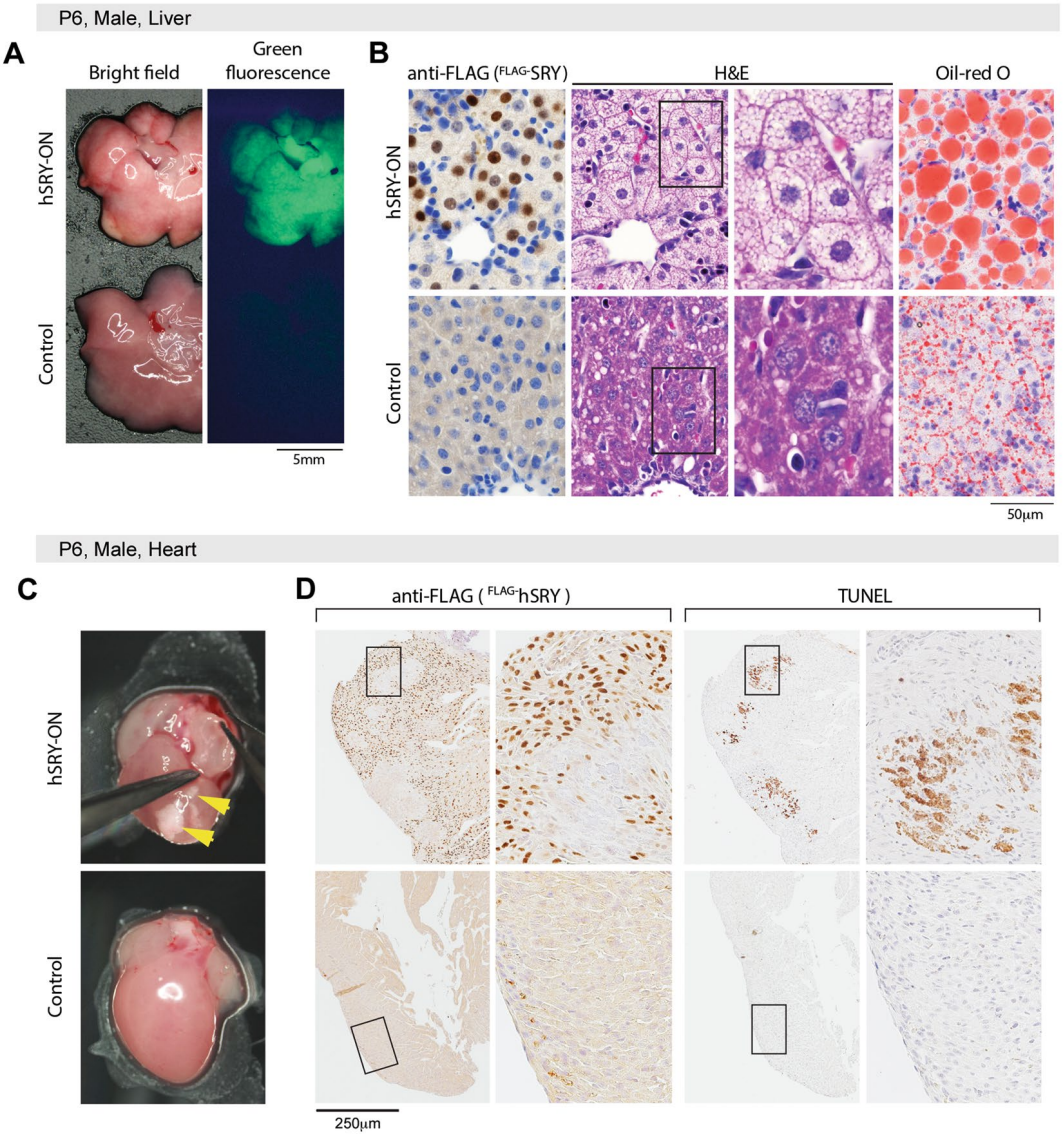
Abnormalities in the liver and heart of hSRY-ON mice. (**A**) Gross morphology of the liver of the hSRY-ON and control pups at P6 stage, showing discolored appearance and green fluorescence expression in a hSRY-ON mouse (top), as compared to control littermate (bottom). (**B**) immunostaining (anti-FLAG, left), H&E (middle) and Oil-red-O staining (right) of liver tissue sections of hSRY-ON (top row) and control littermate (bottom row), showing hSRY expression and hepatic steatosis/fatty liver disease phenotype in hSRY-ON animal. (**C**) White patches/spots (yellow arrowheads) in the heart of an hSRY-ON mouse at P6 age. (**D**) hSRY immunostaining (top, left) and TUNEL staining (top, right) in the heart of hSRY-ON mouse. hSRY protein was expressed in the nuclei of cardiac cells, except those at the TUNEL-positive sites. No hSRY expression or TUNEL staining in the heart of a control animal (bottom). Each boxed area represents the enlarged area in the corresponding immediate right figure.

Analysis of the livers of hSRY^ON^ pups showed that the EGFP was activated (Fig. 2A) and the hSRY protein was expressed specifically in the nuclei of hepatocytes (Fig. 2B, first column), which were also stained negatively in the cytoplasm with hematoxylin and eosin (H&E) (Fig. 2B, second and third columns). Additional staining with Oil Red O showed that the H&E negative regions in the hepatocytes contained significant amount of neutral triglycerides and lipids (Fig. 2B, right column), an indication of hepatic steatosis and non-alcoholic fatty liver disease (NAFLD)^52–54^. The heart of hSRY^ON^ and littermate controls appeared morphologically normal, but selected ones from hSRY^ON^ pups showed white spots/patches (top, Fig. 2C, top), corresponding to likely location(s) of cardiac fibrosis, apoptosis, or myocardial infarction^55^. Immunohistochemistry analysis showed that human SRY was expressed in the nuclei of cardiac cells except those at apoptotic areas (Fig. 2D, left top), which were positive for the TUNEL staining (Fig. 2D, right top). Similar analysis of littermate controls did not show any hSRY expression or reactivity to TUNEL staining (Fig. 2D, lower row).

**Figure 3:**
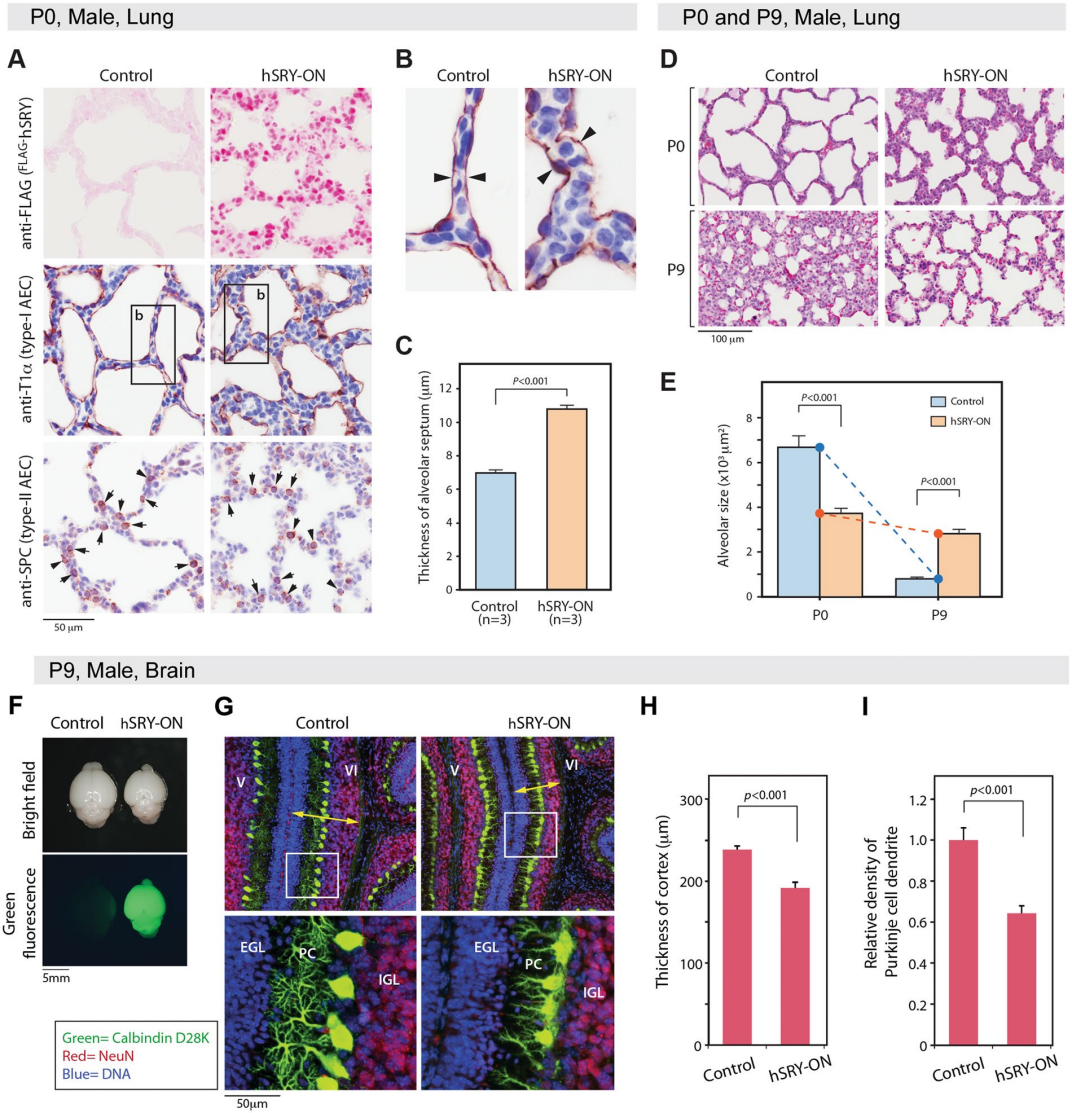
Impaired development of the lung and brain in hSRY-ON mice. (**A**) Immunostaining of hSRY (top), T1α for type I alveolar epithelial cells (middle), and surfactant protein C (SPC) for type II alveolar epithelial cells (bottom) on lung sections of newborn (P0) control (left, column) and hSRY-ON (right, column) mice. (**B**) Enlargements of boxed areas in T1α immunostaining in A. Arrowheads mark the thickness of the alveolar septa. (**C**) Average size of 432 septa thickness from the lung sections of 3 animals of hSRY-ON and control newborn pups. (**D**) H&E staining of lung sections for control (left) and hSRY-ON (right) mice at P0 and P9 age. (**E**) Average size of alveolar sacs from lung sections of 3 animals per group at P0 (350 total measurements) and P9 (550 total measurements) age of hSRY-ON and control mice. Control pups showed normal alveologenesis, starting with large alveolar sacs, which go through microvascular maturation and secondary septa development with reduced overall sac sizes. hSRY-ON pups showed minimal changes in morphology and alveolar sac sizes. (**F**) Gross morphology and green fluorescence expression in brain of an hSRY-ON mouse (right) and control littermate (left). (**G**) Double immunofluorescence of calbindin D28K (green, Purkinje-specific) and neuN (red, nuclear marker for neurons) on cerebella of control (left) and hSRY-ON (right) P9 mice. Yellow arrows indicate the transverse thickness of the molecular, Purkinje and granule cellular layers. Boxed areas on top represent areas of the enlargements in bottom figures. (**H**) Average thickness of cerebellar cortex, and ﻿(**I**) relative density of Purkinje cell dendrites in the cerebella between control and hSRY-ON mice.

The lung development goes through various stages during embryonic and postnatal life^56, 57^. Pups are normally born with alveolar sacs, surfactant production and thinning of the mesenchyme in their lungs to facilitate gas exchanges (Fig. 3A, left control column). They undergo alveologenesis to form secondary septa and microvascular structures to further subdivide the alveolar sacs and increase the alveolar surface for gas exchange and terrestrial life (Fig. 3D, left control column). The hSRY^ON^ pups were born with smaller alveolar sacs and thicker mesenchyme in their lungs (Fig. 3A,B and D, right hSRY-ON columns) and progressed minimally with limited alveologenesis activities, compared to their littermate controls (Fig. 3A,B and D, left control columns). Random measurements of the lung structures at P0 stage showed that the hSRY^ON^ pups have thicker mesenchyme (primary septa) (Fig. 3B and C), but smaller alveolar sac sizes than their control littermates (Fig. 3E). Their alveologenesis seemed to be arrested with minimal increase in secondary septa and microvascular structure formation (Fig. 3D and E). Immunohistochemistry analysis confirmed the abundant expression of the human SRY (Fig. 3A, top right) and presence of type I and II alveolar epithelial cells (AECs), as indicated by immunostaining of T1α and surfactant protein C respectively^58, 59^ (Fig. 3A, middle and bottom), suggesting that ectopic *SRY* expression in the developing lung retarded its postnatal alveologenesis and promoted bronchopulmonary dysplasia, likely resulting in deficiency in alveolar airspace, decrease in gas exchange efficiency, and impairment of respiratory functions^57^.

Parallel to the body sizes, the brains of hSRY^ON^ pups were smaller than those of controls (Fig. 3F). Various neurological stainings, including calbindin D28K immunofluorescence and Golgi-Cox staining, showed that neurogenesis was retarded or impaired in various parts of the brains of these hSRY^ON^ pups, particularly noticeable in the cerebellum (Fig. 3G). The cerebellum undergoes postnatal growth and foliation processes, in which the cerebellar surface is folded into lobules with proliferation of the granule cell precursors, development of the molecular, Purkinje cell and granule cell layers, and arborization of the dendrites, particularly on the Purkinje cells^60^. Such postnatal cerebellar development was significantly impaired in hSRY^ON^ pups, as evidenced by reduced transverse thickness of the molecular, Purkinje cell and granule cell layers (Fig. 3G, yellow arrows, and 3H). Importantly, the size and the dendritic arbors of the Purkinje cells were greatly reduced in hSRY^ON^ animals, as compared to their littermate controls (Fig. 3I). Similar impairments of neurogenesis were also observed in other parts of the brain, such as cerebral cortex-hippocampus-thalamus-hypothalamus regions, using Golgi-Cox staining procedure (Supplemental Fig. 2). These observations suggest an impairing function(s) of an ectopically expressed human *SRY* in the overall neurogenesis in the central nervous system. It is noteworthy that these organ-specific phenotypes, such as impaired neurogenesis in the CNS, cardiomyopathies, deficient alveologenesis, abnormal metabolic homeostasis and hepatic steatosis, are frequently associated with various human diseases, such as autism and schizophrenia^8, 10, 11, 14^, cardiovascular disease^2, 3, 20, 23^, bronchopulmonary dysplasia^16, 57^, non-alcoholic fatty liver disease and hepatocellular carcinoma^17, 18, 30, 61, 62^ respectively, with significant male-biases in incidence, disease penetrance and/or progression in the respective patient populations.

### Ectopic expression of SRY differentially affects the gene regulatory programs and signaling pathways in various organs

**Figure 4:**
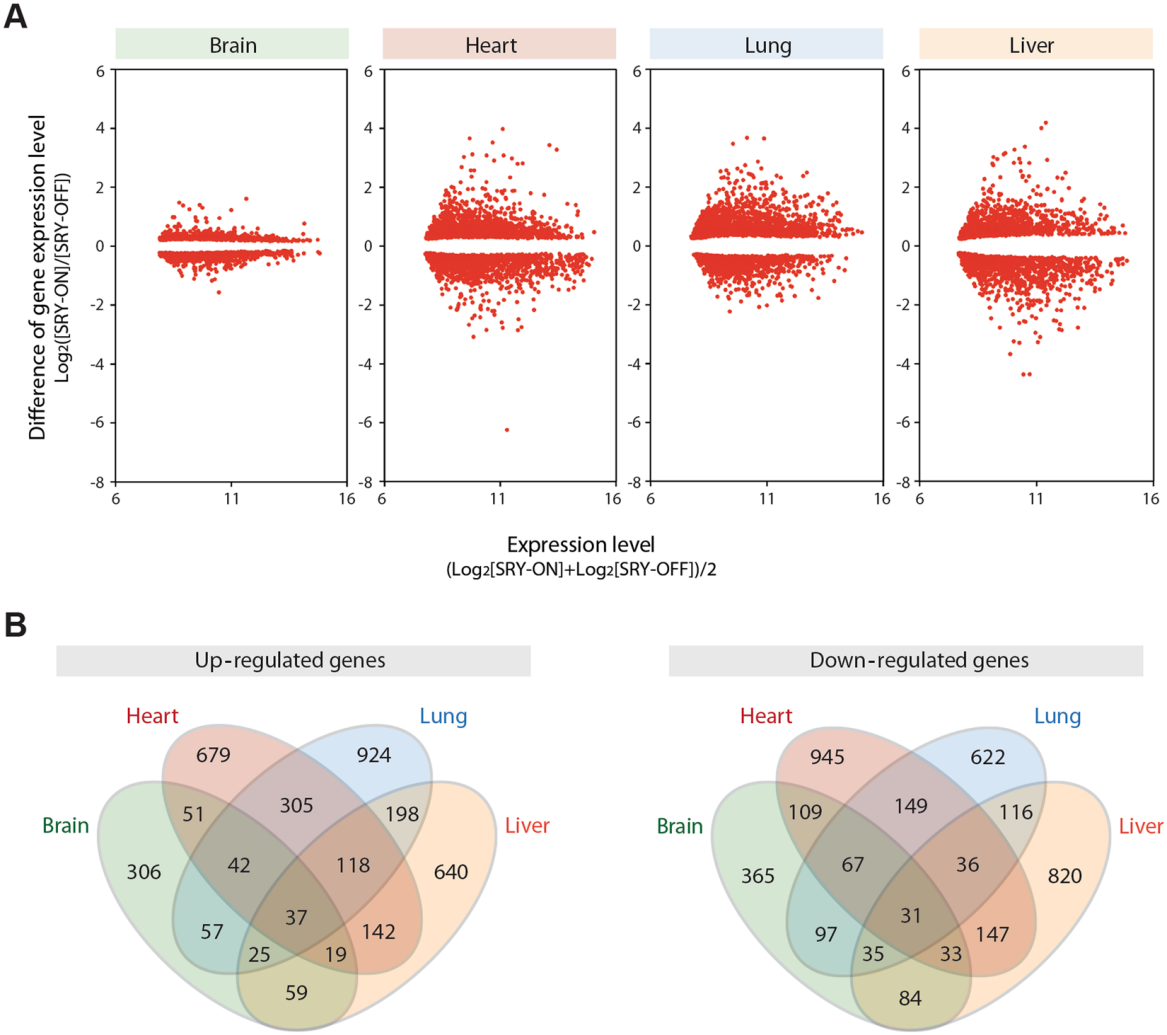
Differential gene expression patterns in brain, heart, lung and liver between hSRY-ON and control (hSRY-OFF) mice at P6 age, as revealed by transcriptome analysis. (**A**) MA plots showing differential gene expression patterns. (**B**) Venn diagrams showing extent of overlaps among differentially up regulated (left) and down regulated (right) genes among the 4 organs examined. See Supplemental Table S1 and S2 for gene lists.

To explore the molecular changes in the brain, heart, lung and liver between hSRY^ON^ and age-matched control pups, we had analyzed their transcriptomes at P6 stage, when close to two-third of the animals were still alive but sufficient growth retardation could be observed (Fig. 1E and F). Transcriptomes were analyzed in biological triplicates between hSRY^ON^ and control pups using the Illumina BeadArray micorarray for the mouse genome^63, 64^. Our results showed that significant changes in gene expression patterns were present at P6 stage between hSRY^ON^ and control pups (Supplemental Table S1). Figure 4A shows the MA plots of the differentially expressed genes between hSRY^ON^ and age-matched controls pups among the 4 organs. The differentially up- and down-regulated genes were compared in Venn diagrams, which showed only minimal overlaps among the 4 organs (Fig. 4B and Supplemental Table S2), suggesting that the ectopically expressed SRY could exert differential effects on the gene expression programs in each organ, examined.

To further deduce the likely signaling pathways and functions affected by *SRY* ectopic expression, the top 700 up- or down-regulated genes and the corresponding differential gene expression levels (log2) from each organ between hSRY^ON^ and control pups were analyzed with the knowledge-base Ingenuity Pathway Analysis (http://www.ingenuity.com/)^45^. The IPA results showed significant numbers of canonical pathways, diseases and functions being affected by ectopic activation of human *SRY* transgene in the mouse. The top canonical pathways included cell cycle control and cholesterol biosynthesis in the brain, mitochondrial dysfunction, oxidative phosphorylation and glycolysis in the heart, FXR/RXR activation, coagulation, and melatonin degradation in the liver, and adhesion, epithelial adherens and cell junction signaling in the lung (Supplemental Table S3). Among the top categories of molecular and cellular functions and physiological system, development and functions, there were several common ones affecting cell growth, cell proliferation, tissue morphology, cell/organismal death, survival and development, which were likely associated with the general growth retardation phenotypes. Various organ-specific categories included the nervous system development and function in the brain, cardiovascular system development in the heart, immune cell trafficking in the lung, and digestive and hepatic system development and function in the liver (Supplemental Table S4). Table 1 shows the top 10 organ-specific and individual diseases and functions, based on their classifications and p-values. In particular, abnormal morphologies of neuritis and neuroglia could be correlated to impaired neurogenesis in the brain while contraction, heart rate, and dilated cardiomyopathy could be associated with the myocardial apoptosis/necrosis or infarction phenotypes in the heart. Abnormal morphology and formation of the pulmonary alveolus could be correlated to the impaired alveologenesis in the lung. For the liver, in addition to morphological abnormalities, hepatic steatosis and necrosis could contribute to the non-alcoholic fatty liver disease phenotypes. Notably, various tumors, e.g. glioma and gliobastoma, lung cancer, and hepatocellular carcinoma, seem to be present among the annotated diseases and functions in the brain, lung and liver respectively, suggesting that ectopic expression of *hSRY* could predispose these organs to oncogenesis, if the animals were to survive to adulthood. Collectively, the results from the transcriptome and IPA analyses support the notion that ectopic activation of the human *SRY* transgene retards general growth activities but differentially affects the developmental processes, physiological functions and likely oncogenic predisposition of various organs in the hSRY^ON^ animals.

**Table 1:** Individual diseases or function annotation pertaining to the specific organs and phenotypes.

Organ	Diseases or Functions Annotation	Categories	p-value	# Molecules
**Brain**	**Top 10 organ-related diseases and functions**			
	Abnormal morphology of central nervous system	Nervous System Development and Function, Neurological Disease	2.56E-08	46
	Glioma cancer	Cancer, Neurological Disease, Organismal Injury and Abnormalities	7.60E-08	38
	Tauopathy	Neurological Disease, Organismal Injury and Abnormalities, Psychological Disorders	1.29E-07	53
	Morphology of central nervous system	Nervous System Development and Function	1.73E-07	50
	Morphology of nervous system	Nervous System Development and Function	2.16E-07	71
	Abnormal morphology of neurites	Cell Morphology, Nervous System Development and Function, Neurological Disease, Tissue Morphology	2.22E-07	20
	Glioblastoma cancer	Cancer, Neurological Disease, Organismal Injury and Abnormalities	3.61E-07	36
	Morphology of neurites	Cell Morphology, Nervous System Development and Function, Tissue Morphology	5.56E-07	25
	Abnormal morphology of nervous system	Nervous System Development and Function, Neurological Disease	1.12E-06	59
	Morphology of neuroglia	Cell Morphology, Nervous System Development and Function	1.60E-06	16
**Heart**	**Top 10 organ-related diseases and functions**			
	Function of cardiovascular system	Cardiovascular System Development and Function	3.34E-18	55
	Heart rate	Cardiovascular System Development and Function	1.32E-16	46
	Morphology of cardiovascular system	Cardiovascular System Development and Function	1.67E-15	78
	Hypertrophy of heart	Cardiovascular Disease, Developmental Disorder, Organismal Injury and Abnormalities	9.76E-15	49
	Contraction of heart	Cardiovascular System Development and Function, Organ Morphology	3.00E-14	33
	Morphology of muscle cells	Cell Morphology, Organ Morphology, Skeletal and Muscular System Development and Function, Tissue Morphology	5.84E-14	40
	Morphology of heart	Cardiovascular System Development and Function, Organ Morphology, Organismal Development	2.65E-13	60
	Contraction of cardiac muscle	Cardiovascular System Development and Function, Organ Morphology, Skeletal and Muscular System Development and Function	7.20E-13	23
	Dilated cardiomyopathy	Cardiovascular Disease, Organismal Injury and Abnormalities, Skeletal and Muscular Disorders	7.53E-13	36
	Mass of heart	Cardiovascular Disease, Cardiovascular System Development and Function, Organ Morphology, Organismal Development	2.28E-12	26
**Lung**	**Top 10 organ-related diseases and functions**			
	Lower respiratory tract disorder	Respiratory Disease	3.06E-13	45
	Morphology of respiratory system	Respiratory System Development and Function	1.65E-09	39
	Morphology of respiratory tract	Respiratory System Development and Function	2.74E-09	30
	Abnormal morphology of respiratory system	Respiratory Disease, Respiratory System Development and Function	3.01E-09	37
	Lung tumor	Cancer, Organismal Injury and Abnormalities, Respiratory Disease	3.50E-09	141
	Respiratory system tumor	Cancer, Organismal Injury and Abnormalities, Respiratory Disease	4.98E-09	143
	Abnormal morphology of lung	Embryonic Development, Organ Development, Organ Morphology, Organismal Development, Organismal Injury and Abnormalities, Respiratory Disease, Respiratory System Development and Function, Tissue Development	7.78E-09	26
	Morphology of lung	Embryonic Development, Organ Development, Organ Morphology, Organismal Development, Respiratory System Development and Function, Tissue Development	1.35E-08	27
	Formation of lung	Embryonic Development, Organ Development, Organismal Development, Respiratory System Development and Function, Tissue Development	2.22E-08	35
	Abnormal morphology of pulmonary alveolus	Embryonic Development, Organ Development, Organ Morphology, Organismal Development, Organismal Injury and Abnormalities, Respiratory Disease, Respiratory System Development and Function, Tissue Development	2.83E-08	19
**Liver**	**Top 10 organ-related diseases and functions**			
	Morphology of liver	Digestive System Development and Function, Hepatic System Development and Function, Organ Morphology, Organismal Development	4.88E-17	53
	Hepatic steatosis	Gastrointestinal Disease, Hepatic System Disease, Metabolic Disease, Organismal Injury and Abnormalities	1.40E-15	47
	Necrosis of liver	Cell Death and Survival, Gastrointestinal Disease, Hepatic System Disease, Organismal Injury and Abnormalities	3.65E-11	35
	Abnormal morphology of liver	Digestive System Development and Function, Gastrointestinal Disease, Hepatic System Development and Function, Hepatic System Disease, Organ Morphology, Organismal Development, Organismal Injury and Abnormalities	7.15E-11	34
	Abnormal morphology of hepatobiliary system	Digestive System Development and Function, Gastrointestinal Disease, Hepatic System Development and Function, Hepatic System Disease, Organismal Development, Organismal Injury and Abnormalities	9.47E-11	35
	Hepatocellular carcinoma	Cancer, Gastrointestinal Disease, Hepatic System Disease, Organismal Injury and Abnormalities	8.65E-09	69
	Mass of liver	Digestive System Development and Function, Hepatic System Development and Function, Organ Morphology, Organismal Development	1.03E-08	21
	Proliferation of liver cells	Cellular Development, Cellular Growth and Proliferation, Digestive System Development and Function, Hepatic System Development and Function, Organ Development	1.26E-08	26
	Morphology of liver cells	Cell Morphology, Digestive System Development and Function, Hepatic System Development and Function, Organ Morphology, Organismal Development	1.39E-07	17
	Morphology of hepatocytes	Cell Morphology, Digestive System Development and Function, Hepatic System Development and Function, Organ Morphology, Organismal Development, Tissue Morphology	1.74E-07	16

## Discussion

SRY is absolutely required for normal testis differentiation, but perhaps not critical in development and/or physiology of other organs/cell types in mammals. Our study was initially inferred from the observations that SRY and SOX9 share a significant number of common target genes, and are capable of binding to the promoters and affecting their expressions^45^, suggesting that ectopic *SRY* expression in non-gonadal cells, as observed frequently in various somatic tissues^25–29^, could compete with the normal gene regulatory functions of the resident *SOX* genes. The establishment of a Cre-LoxP transgene activation system has provided an experimental strategy to examine the effects of such aberrant *hSRY* expression in whole animals. As an initial attempt, we have specifically and globally activated the human *SRY* transgene at the single-cell embryonic stage in the mouse. The selection of the human *SRY* gene in this evaluation was based on two reasons. First, beside their SRY-related HMG box (SOX) domain, the human *SRY* and mouse *Sry* gene diverged considerably at the flanking sequences^65, 66^. The mouse *Sry* has evolved to encode a glutamine-rich domain at its carboxyl terminus, absent in the *SRY/Sry* of most other mammals^65^. This glutamine-rich domain is required for proper determination of the male sex in the mouse^67^. Since we are modeling human diseases, we have selected the human *SRY* for evaluation of its effects in the development and physiology of transgenic mice. Second, early studies showed that the human *SRY* is incapable of mediating any sex reversal in transgenic XX mice^68^, thereby eliminating the complication of gonadal dysgenesis, abnormal actions of the sex hormones and their biological consequences in the system. Our results clearly support the hypothesis that activation of the human *SRY* on non-gonadal cells could exert disruptive effects on the developmental and physiological processes in multiple organs. We surmise that such genetic modifying actions of the *hSRY* transgene were likely to have fetal origins and initiated in early stages during embryogenesis^69, 70^. Subsequent characterization of the animals showed that the major organs, such as the thymus, brain, heart, lung, digestive tract and liver, were greatly affected by such aberrant SRY actions, resulting in inhibited thymus differentiation, impaired neurogenesis, cardiac necrosis and apoptosis, arrested pulmonary alveologenesis, and hepatic steatosis and non-alcoholic fatty liver disease. Although the exact reasons for their postnatal lethality is currently uncertain, these impairments and insufficiencies could be intertwined and collectively contribute to the observed phenotypes in the various organs. For example, impaired thymus development could affect the T-cell differentiation and compromise immune system; deficiency in neurogenesis of the CNS could affect the innate suckling activities and other important neural functions; retarded pulmonary alveologenesis could affect the respiratory functions and oxygen supply to the brain and other vital organs; necrosis and apoptosis of cardiac cells could weaken the heart and impair the circulatory system and supplies of nutrients; and hepatic steatosis could minimize metabolism and detoxification functions of the liver. Collectively these abnormalities could contribute to the postnatal growth retardation and lethality in a multi-organ failure mechanism(s).

At present, the molecular mechanisms of SRY modifying actions are uncertain. Our initial postulation suggests that SRY could compete with the resident SOX transcription factor(s) and disrupt its/their gene regulatory programs^36, 38, 40, 42, 44, 45^. However, we believe that this could be one of many possible mechanisms of SRY modulatory actions. Detailed characterization of two SRY targets, the monoamine oxidase A (*MAOA*) and *RET* oncogene, involved in neural development/cognitive functions and enteric nervous system development respectively^9, 28, 71, 72^, show that SRY could bind to the promoters of both genes, but exert its modifying actions with different mechanisms. SRY collaborates with the transcription factor SP1, and up regulates the endogenous *MAOA* expression at both transcription and protein levels in neural cells^73^. SRY exerts its modulating functions on *RET* expression by competing and interfering the interactions between the related SOX10 and two resident transcription factors, i.e. NKx2–1 and PAX3, on the *RET* promoter, thereby repressing their transcriptional activation of *RET* gene^28^. SRY effects on *MAOA* and *RET* expression could affect normal synaptogenesis and neurotransmission, and enteric nervous system development, and contribute to pathogeneses of diseases associated with these two genes, i.e. depression/cognitive disorders and Hirschsprung disease respectively. Significantly, these disorders show various sexual dimorphisms in disease incidence, susceptibility and penetrance among the respective patient populations^9, 71, 72, 74^. Hence, these studies support the notion that *SRY* could affect its target gene expression through different interactive partners and molecular mechanisms. Indeed, various studies show that SRY and SOX proteins interact with a variety of co-factors, and form complexes with specific transcriptional functions and propensity^75, 76^. SRY interacts with several transcription activators, such as SF1 and SP1; chromatin modulator, such as the poly(ADP-ribose)polymerase 1 (PARP1); transcriptional repressors, such as KAP1-HP1 gene-silencing complex; β-catenin in the WNT signaling pathway; the male hormone receptor, i.e. androgen receptor; and partnering NKx2.1 and PAX3 transcription factors, as in the case of *RET* gene regulation, and differentially modulate the expression of respective target genes^28, 73, 77–82^. Hence, the genetic modifying effects of an ectopically expressed *SRY* could be extremely complex; and its stimulatory/disruptive actions are context-specific and dependent on availability of co-factors in the affected cells/tissues in spatiotemporal manners.

SRY contributions to sexual dimorphisms on human development and diseases depend on at least two aspects under natural conditions. First, the Y-located endogenous *SRY* gene needs to be epigenetically activated in the tissue or cell types being affected. Previous studies demonstrated that *SRY* expression could be detected in various tissues apart from the testis, such as the brain, enteric nervous system, kidney and other organs/cell types under normal and diseased conditions^26, 28, 29, 83, 84^. Hence, such ectopic expressions of *SRY* in non-gonadal cells/tissues could be likely events. Currently, the mechanisms for such epigenetic activation of the Y-located *SRY* are still uncertain. Presumably, various biological and physical environments both at embryonic and postnatal stages could influence such non-gonadal *SRY* expression, the nature and mechanisms of which need to be further elucidated. Second, the spatiotemporal sites and the magnitudes of such activation of the Y-located *SRY* could be key in mediating the biological consequences. Further, some cell types/organs could harbor the specific co-factors important for *SRY* to exert its genetic modifying actions while others could be deficient in such co-factors, thereby resulting in differential actions and variable effects. We surmise that under mild activation state *SRY* could exert normal sexual dimorphisms, such as brain structures and blood pressure regulation between the sexes^2, 3, 5^, while abnormal and elevated levels of activation could result in sexually dimorphic diseases, such as autism spectrum disorder and hypertension respectively for examples, with significant male incidence and penetrance^7, 8, 85^. Accordingly, the biological effects of *SRY* in sexual dimorphisms between the sexes are dependent on how, when, where and how much it is aberrantly activated during the different stages of the life of a male individual. The present study has established and demonstrated the feasibility of a Cre-LoxP transgene activation system, thereby providing an experimental strategy using tissue-specific and/or developmental *Cre* transgenic lines to functionally evaluate the contributions of *SRY* and other MSY genes in health and diseases of man.

## Methods

### Animals

The *Signalox-hSRY* transgene vector was constructed as described in the supplemental Materials and Methods. The *Signalox-hSRY* mouse line was generated by using TARGATT technology^47^ at Applied StemCell Inc. (Milpitas, CA), to integrate a single copy of *Signalox-hSRY* transgene into the *H11* locus (Fig. 1A) on chromosome 11 of the mouse genome. All mice in the present study were kept in the FVB/N genetic background. The *Signalox-hSRY* mice without Cre-mediated recombination are designated as hSRY-OFF (or ﻿hSRY^OFF^), to indicate that *hSRY* was not activated. *Ddx4-Cre* mouse line was obtained from the Jackson Laboratory (Bar Harbor, ME). The fully recombined *Signalox-hSRY* transgenic mice were obtained from crosses between heterozygous male *Signalox-hSRY* mice and female *Ddx4-Cre* mice (Fig. 1A), and are designated as hSRY-ON (or ﻿hSRY^ON^). Since the Cre recombinase was transferred from the cytoplasm of the oocytes and activated the *Signalox-hSRY* transgene in the single-cell embryos irrespective the transgenic status of *Ddx4-Cre* transgene, littermates without *Signalox-hSRY* transgene, i.e. either non-transgenic or transgenic for only *Ddx4-Cre*, are designated as control littermates. The mouse genotype was screened by PCR on genomic DNA from tail biopsy using primer sets as following: *Cre*, 5′-CCA CGA CCA AGT GAC AGC AAT G-3′ and 5′-CAG AGA CGG AAA TCC ATC GCT C-3′; *hSRY*, 5′-GAA CGC ATT CAT CGT GTG GTC-3′ and 5′-CCA TTC TTG AGT GTG TGG CTT TC-3′; *DsRed*, 5′-TCC AAG GTG TAC GTG AAG CAC C-3′ and 5′-GGA CTT GAA CTC CAC CAG GTA GTG-3′; hSRY-ON (after recombination), 5′-GCC TCT GCT AAC CAT GTT CAT GC-3′ and 5′-CCA TTC TTG AGT GTG TGG CTT TC-3′. Fluorescent images of raw tissues were recorded by using a Leica MZFLIII-DC300F digital imaging system.

All animals were maintained at the Animal Care Facility of San Francisco VA Medical Center. The Institutional Animal Care and Use Committee approved all experimental procedures in accordance with the NIH *Guide for Care and Use of Laboratory Animals*.

### Western blot analysis

Western blot analysis was performed as described previously^46, 86^ with anti-FLAG mouse IgG (clone M2, Sigma-Aldrich) and anti-actin mouse IgG (clone C4, EMD Millipore), and detected by IRDye680-conjugated anti-mouse IgG antibodies (LI-COR, Lincoln, NE), and infrared imaging system Odyssey (LI-COR).

### Immunofluorescence and Immunohistochemical analysis

Immunofluorescence and immunohistochemical analyses of tissue sections were performed as described previously^30, 46^. Antibodies specific to the following proteins were obtained from various vendors, as indicated: anti-FLAG mouse IgG (clone M2, Sigma-Aldrich), anti-Calbindin D28K goat polyclonal IgG (C-20, Santa Cruz Biotechnology, Dallas, TX), anti-NeuN mouse IgG (clone A60, EMD Millipore), anti-Prosurfactant Protein C rabbit antiserum (EMD Millipore), and anti-Podoplanin hamster IgG (clone 8.1.1, Developmental Studies Hybridoma Bank, Iowa, IA). For the immunofluorescence analyses, Alexa Fluor 594 (red)-conjugated anti-mouse IgG and Alexa Fuor 488 (green)-conjugated anti-goat IgG (Molecular Probes/Thermo Fisher Scientific) were used as secondary antibodies. Nuclei were visualized by staining with 4′,6-Diamidine-2′-phenylindole dihydrochloride (DAPI). Immunofluorescence was examined with a Nikon Eclipse Ti inverted microscope and digital imaging system. For the immunohistochemical analyses, the immunoreactive sites were detected with the SuperPicture polymer detection kit for mouse IgG (ZYMED/Invitrogen, Carlsbad, CA) or VECTASTAIN ABC-Elite HRP kit for hamster IgG (Vector laboratories). Sections were counterstained by hematoxylin to visualize the nuclei (Abcam, Cambridge, MA), and examined and recorded with a Zeiss Axio Imager A2 digital imaging system. Area quantification was performed with the ImageJ program (Rasband, W.S., ImageJ, U. S. National Institutes of Health, Bethesda, Maryland, USA, http://imagej.nih.gov/ij/, 1997–2016).

Terminal deoxynucleotidyl transferase dUTP nick end labeling (TUNEL) was performed with the ApopTag peroxidase *in situ* apoptosis detection kit (S7100, EMD Millipore) according to the manufacture’s instructions.

### Oil Red O staining

Oil Red O staining was performed as described^54^. In brief, the dissected liver tissue was frozen in liquid nitrogen and sectioned at 12 μm with a cryostat (CM1850, Leica Biosystems, Buffalo Grove, IL). After drying for 10 min at room temperature, sections were stained by 3.75 g/L Oil Red O (Sigma-Aldrich) dissolved in 60% isopropanol-water solution for 5 min, and washed with water for 30 min. Sections were counterstained by hematoxylin to visualize the nuclei.

### Golgi-Cox Impregnation

Golgi-Cox impregnation (Golgi staining) of neural tissues were performed on the brain tissues of P9 and P12 ﻿hSRY^ON^ and control pups, using a FD Rapid GolgiStain kit (FD Neurotechnologies, Inc., Columbia, MD), according to recommended protocols of the manufacturer^87^. After Golgi staining, 100 to 200 micron sections were obtained across the brain, and mounted on microscope slides without counterstaining. The tissue sections were examined and recorded with the Zeiss Axio Imager 2 microscope and digital image recording system, as above.

### Lung alveolar septa and alveolar space, and cerebellum cortex and Purkinje cell dendrite measurements

The thickness of the alveolar septum in the lungs between hSRY-ON and control pups at P0 stage was measured randomly in 3 male animals in each group and approximately 144 measurements per animal. The alveolar space sizes were measured similarly at P0 and P9 stages with approximately 120 and 190 measurements per animal respectively. The thickness of the lobe-VI in cerebellum cortex was measured at 6 sites in 3 mice per group with the immunofluorescence images. The densities of the Purkinje cell dendrites were measured within a 105-micron x 20-micron area at 18 sites in 3 control and 14 sites in 3 hSRY-ON mice. The measurements for the alveolar space and the dendrite density were performed with the NIH Image J program. The means and standard errors were calculated from the respective measurements between hSRY-ON and control pups and the p-values were analyzed with the Student’s t-test. P-values ≤ 0.05 were considered to be statistically significant.

### Transcriptome analyses

Total RNAs were isolated from the dissected tissues of male hSRY-OFF and hSRY-ON mice (n = 3 for each group) at postnatal age day 6 (P6) with the TRIZOL Plus RNA Purification kit (Ambion/Thermo Fisher Scientific). To adjust the genetic background in the transcriptome analyses, we selected the hSRY-ON mice that did not harbor the *Ddx4*-*Cre* transgene. The global gene expression analyses were carried out with MouseRef-8 v2.0 expression BeadChip (Illumina, San Diego, CA), a microarray-hybridization based method, at UCLA Neuroscience Gnomic Core (Los Angels, CA). Normalization and differential gene expression analyses were performed with an R package TCC^88^. The top 700 differentially expressed genes of each organ were analyzed with the Ingenuity Pathways Analysis using the core analysis suite in November 2016.

## Electronic supplementary material

Supplemental Information

Supplemental Table S1

Supplemental Table S2

Supplemental Table S3

Supplemental Table S4
