# Supplementary material for: Aberrant activation of the human sex-determining gene in early embryonic development results in postnatal growth retardation and lethality in mice
Source: Sci Rep. 2017 Jun 23;7:4113. doi: 10.1038/s41598-017-04117-6 (PMC5482865; doi:10.1038/s41598-017-04117-6)
Supplement: Supplementary file 1 — Supplemental Information [file 41598_2017_4117_MOESM1_ESM.pdf]

Aberrant activation of the human sex-determining gene in early embryonic development results in postnatal growth retardation and lethality in mice

Tatsuo Kido, Zhaoyu Sun and Yun-Fai Chris Lau*

Department of Medicine
VA Medical Center
Institute for Human Genetics
University of California, San Francisco
San Francisco, California

Corresponding Author: chris.lau@ucsf.edu

SUPPLEMENTAL INFORMATION

Supplemental Information

Materials and Methods

Construction of the *Signalox-hSRY* transgene vector. All PCRs for vector construction were performed by using the FastStart High Fidelity PCR system (Roche Diagnostics, Indianapolis, IN) according to the manufacture's instructions. The sequences of PCR products were verified by DNA sequencing after cloning into *pGEM-Teasy* vector (Promega, Madison, WI).

pGEM-LoxP-DsRed: LoxP sequences were added at the 5' end of DsRed2-polyA cassette of *pDsRed2-N1* (Clontech, Mountain View, CA) by PCR, and cloned into the cloning site of *pGEM-Teasy* vector. The primer sequences were 5'-GCC CAT GGA TAA CTT CGT ATA GCA TAC ATT ATA CGA AGT TAT GCC ACC ATG GCC TCC TCC GAG AAC-3' and 5'-GCA GAT CTA TGC AGT GAA AAA AAT GCT TTA TT-3'. Underline indicates the LoxP sequences.

pBT378-LoxP-DsRed: The DNA fragment of LoxP-DsRed-polyA was extracted from *pGEM-LoxP-DsRed* vector by using SacI and SacII, and treated by Klenow enzyme (Promega) to generate blunt-ends. *pBT378* vector (Applied StemCell, Milpitas, CA), harboring attBs for ϕC31-mediated cassette exchange, was digested by using PstI and EcoRI. After treatment with Klenow enzyme to generate blunt-ends, pBT378 vector was ligated with the DNA fragment of LoxP-DsRed-polyA.

pBT378-CAGp-LoxP-DsRed: The DNA fragment of the ubiquitously active chicken β-actin promoter (CAGp) was extracted from *pCAGGS-NLS-Cre-PGKPuro/cg* vector (a gift of C. Lobe, Sunnybrook and Women's College Health Science Center, Toronto) by using SalI and PstI, and inserted into the HindIII site of *pBT378-LoxP-DsRed* vector after Klenow treatment. Procedures are graphically demonstrated as Diagram A, below.

pGEM-FLAG-hSRY: The gene coding FLAG-tagged human SRY (FLAG-hSRY) (1) flanked by XhoI sites at both 5' and 3' ends was cloned into the cloning site of *pGEM-Teasy* vector.

pGEM-LoxP-EGFP: LoxP sequences were added at the 5' end of IRES-EGFP cassette of *pIRES2-EGFP* (Clontech, Mountain View, CA) by PCR, and cloned into the cloning site of *pGEM-Teasy* vector. The primer sequences were 5'-GCA GAT CTA TAA CTT CGT ATA GCA TAC ATT ATA CGA AGT TAT CTC GAG CTC AAG CTT CGA ATT CTG-3' and 5'-GCG AAT TCT TAC TTG TAC AGC TCG TCC ATG CC-3'. Underline indicates the LoxP sequences.

pGEM-LoxP-hSRY-EGFP: The DNA fragment of FLAG-hSRY was extracted from *pGEM-FLAG-hSRY* by using XhoI, and inserted into the XhoI site of *pGEM-LoxP-EGFP* vector.

pBT378-CAGp-LoxP-DsRed-LoxP-hSRY-EGFP (pSignalox-hSRY): *pBT378-CAGp-LoxP-DsRed* was digested by BlgII and treated with Klenow enzyme. The cassette of LoxP-hSRY-EGFP was extracted from *pGEM-LoxP-hSRY-EGFP* vector by using NotI, treated by Klenow enzyme, and inserted into the BglII site as prepared above. The resultant vector is designated as *pSignalox-hSRY* in the present study. Procedures are graphically demonstrated as Diagram B and C, below.

References

1. Oh, H.J., Li, Y. and Lau, Y.F. (2005) Sry associates with the heterochromatin protein 1 complex by interacting with a KRAB domain protein. *Biol. Reprod.*, **72**, 407-415.

A.

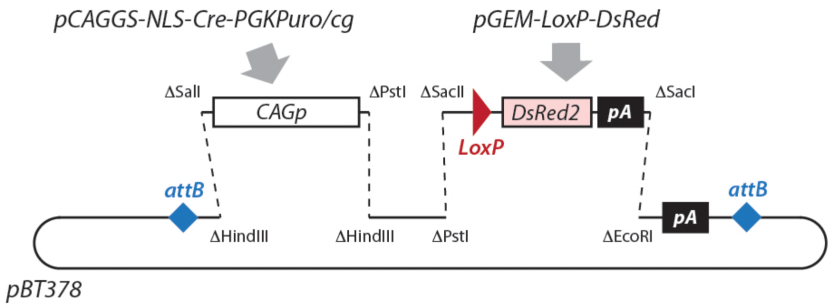

Construction of *pBT378-CAGp-LoxP-DsRed* vector

B.

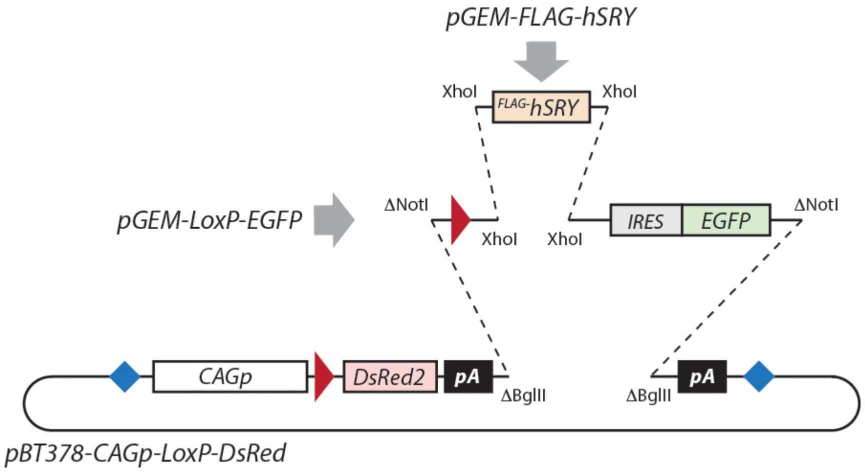

Construction of *pBT378-CAGp-LoxP-DsRed* vector

C.

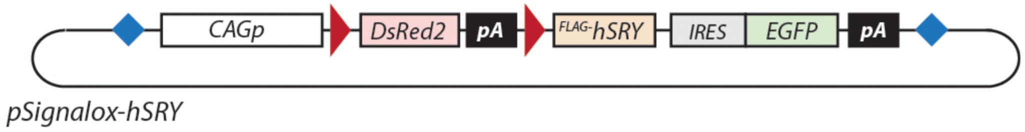

pSignalox-hSRY vector

Figure A-C. Illustrations of cloning strategy for Signal-hSRY vector.

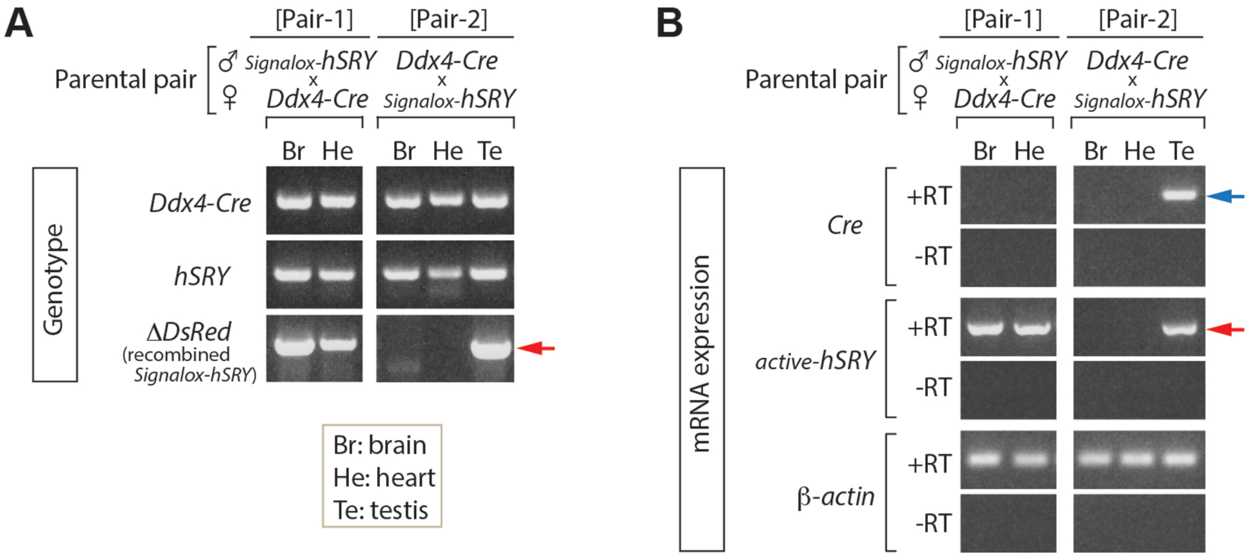

Supplemental Figure 1. Parental-pair dependent recombination and expression of the *Signalox-hSRY* transgene mediated by *Ddx4-Cre*. A P6 male offspring from a parental pair of a male S*ignalox-hSRY* mouse and a female D*dx4-Cre* mouse (pair-1), and a P36 male offspring from a parental pair of a male D*dx4-Cre* mouse and a female S*ignalox-hSRY* mouse (pair-2) were analyzed with PCR and RT-PCR for the genome recombination and transgene expression respectively. (A) Genotype was analyzed with PCR on DNAs from the brain, Br; heart, He; and testis, Te, as described in the Methods. Results showed that S*ignalox-hSRY* transgene was recombined in all organs of the offspring from pair-1, but was recombined only in the testis of the offspring from pair-2 (ΔDsRed, arrow). (B) Results of RT-PCR analysis of RNAs from the same organs to detect the expression of transgene, using the Roche Transcriptor Reverse Transcriptase kit. Cre was expressed only in testis (blue arrow). The transcript from the recombined *Signalox-hSRY* (active-h*SRY)* was expressed in all organs of the offspring from pair-1, but it was expressed only in the testis of the offspring from pair-2 (red arrow). β-actin was used as a positive control.

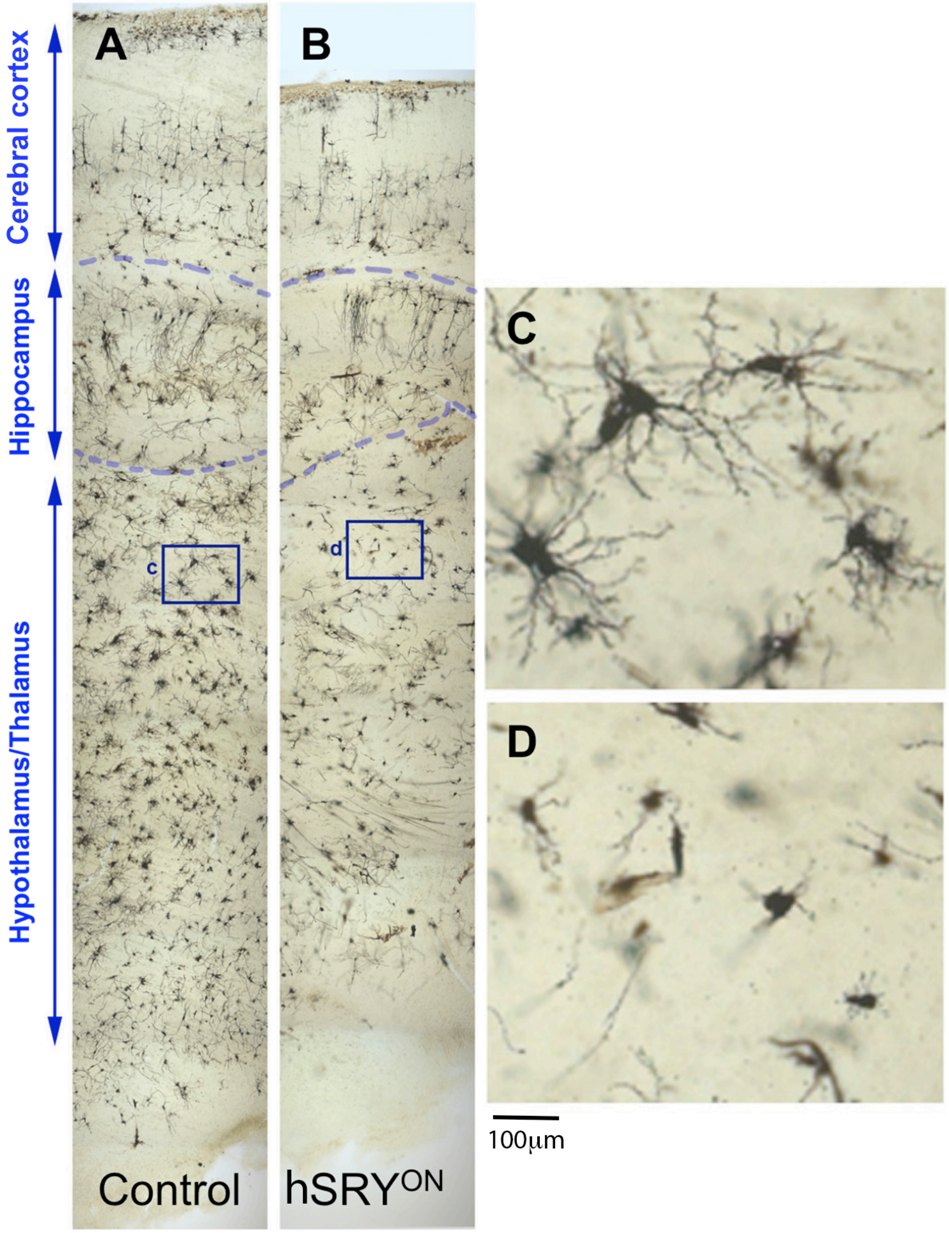

Supplemental Figure 2. Golgi-Cox staining of sagittal sections across the cerebral cortex, hippocampus and thalamus/hypothalamus areas in the brains of a control littermate (A) and a hSRY^{ON} mouse (B) at P12 postnatal stage. C and D are enlargements of boxed areas in A and B respectively, showing reduced arborization of dendrites in the thalamus area in the brain of the hSRY^{ON} mouse (D).
